# Topological Progression in Proliferating Epithelia Is Driven by a Unique Variation in Polygon Distribution

**DOI:** 10.1371/journal.pone.0079227

**Published:** 2013-11-05

**Authors:** Daniel Sánchez-Gutiérrez, Aurora Sáez, Alberto Pascual, Luis M. Escudero

**Affiliations:** 1 Instituto de Biomedicina de Sevilla, Hospital Universitario Virgen del Rocío/CSIC/Universidad de Sevilla, Seville, Spain; 2 Escuela Técnica Superior Ingeniería, Universidad de Sevilla, Seville, Spain; 3 Centro de Investigación Biomédica en Red de Enfermedades Neurodegenerativas (CIBERNED), Madrid, Spain; University of Bern, Switzerland

## Abstract

Morphogenesis is consequence of lots of small coordinated variations that occur during development. In proliferating stages, tissue growth is coupled to changes in shape and organization. A number of studies have analyzed the topological properties of proliferating epithelia using the *Drosophila* wing disc as a model. These works are based in the existence of a fixed distribution of these epithelial cells according to their number of sides. Cell division, cell rearrangements or a combination of both mechanisms have been proposed to be responsible for this polygonal assembling. Here, we have used different system biology methods to compare images from two close proliferative stages that present high morphological similarity. This approach enables us to search for traces of epithelial organization. First, we show that geometrical and network characteristics of individual cells are mainly dependent on their number of sides. Second, we find a significant divergence between the distribution of polygons in epithelia from mid-third instar larva versus early prepupa. We show that this alteration propagates into changes in epithelial organization. Remarkably, only the variation in polygon distribution driven by morphogenesis leads to progression in epithelial organization. In addition, we identify the relevant features that characterize these rearrangements. Our results reveal signs of epithelial homogenization during the growing phase, before the planar cell polarity pathway leads to the hexagonal packing of the epithelium during pupal stages.

## Introduction

Epithelia are one of the fundamental units of animal development. These tissues undergo cell shape changes and reorganizations within the epithelial plane that sculpt the final organism [1], [2]. Remodeling is a finely controlled process that involves different types of rearrangements. Gradually, small reorganizations vary the topology of the whole epithelia. Despite being a highly dynamic process, it also has to be ordered and fairly reproducible enabling the correct formation of mature organs with a cellular assembly suited to their specialised functions. Combination of genetics and imaging techniques has enabled profound advances in the understanding of fast and dramatic morphogenetic events in *Drosophila*. Clear examples of them are the rearrangements induced during convergent extension or ommatidia rotation, the tissue remodeling dependent on cell apical constriction or the zippering of the embryo during the process of dorsal closure [3], [4], [5], [6], [7], [8], [9], [10], [11], [12], [13].

The wing primordium has been particularly well studied in terms of the genetic inputs that drive its development. This great understanding has made its monolayer epithelium the perfect target to analyze the development of epithelial topology. During the four days of larval development, wing imaginal disc grows from 20 to approximately 50000 cells [14], [15]. Later in development, at pupa stages, cell divisions stop and this epithelium became an almost perfect hexagonal lattice that will originate the adult wing. Interestingly, through this period of intense proliferation the topology of the epithelium seems heterogeneous with not apparent order or governing organizational rules. Several groups have tried very different approaches to understand the mechanisms of cells packing during the development of the wing disc. Remarkably, all of them are based directly or indirectly in the number of sides of the cells and detected similar values of the polygon distribution in proliferating wing discs [1], [2], [16], [17], [18], [19], [20], [21], [22], [23], [24]. Different approaches have been used so far to understand the basis of the emergence of epithelial topology and cellular packing. They goes from genetic analysis of morphological changes [19], the analysis of the interplay between of proliferation, mitotic cleavage and topology [17], [23], [25], computational modelling, [21], [22] or the study of the biophysical properties of the cells proposing a vertex model [16], [24]. These studies imply the existence of a proliferative phase not involved in organization and a second non-proliferative phase (after pupa formation) where final order is acquired.

Here we try to address if the proliferative phase can already contribute to epithelial organization, and if that is the case, to define what are the main organizational clues that arise during this early developmental time point. In order to do that we have performed for the first time a detailed comparison between two developmental points during the proliferative phase: mid-third instar larva and early prepupa (separated only by 24 hours of development). We demonstrate the existence of differences between these two close stages of development using several systems biology methods (including our new network-based image analysis approach, [26], [27], [28] and a high number of samples (31 samples and a total of 15951 cells). The network characteristics that discriminate between these two stages provide also new biological information about this developmental process. Using these methods, we are able to detect the emergence of homogeneity and regularity before the end of the larval proliferative stage. This represents the first hints of hexagonal packing that will occur on the pupal wing disc.

## Results

### Computerized analysis shows that cellular characteristics do not vary between larval and prepupal samples

**Figure 1:**
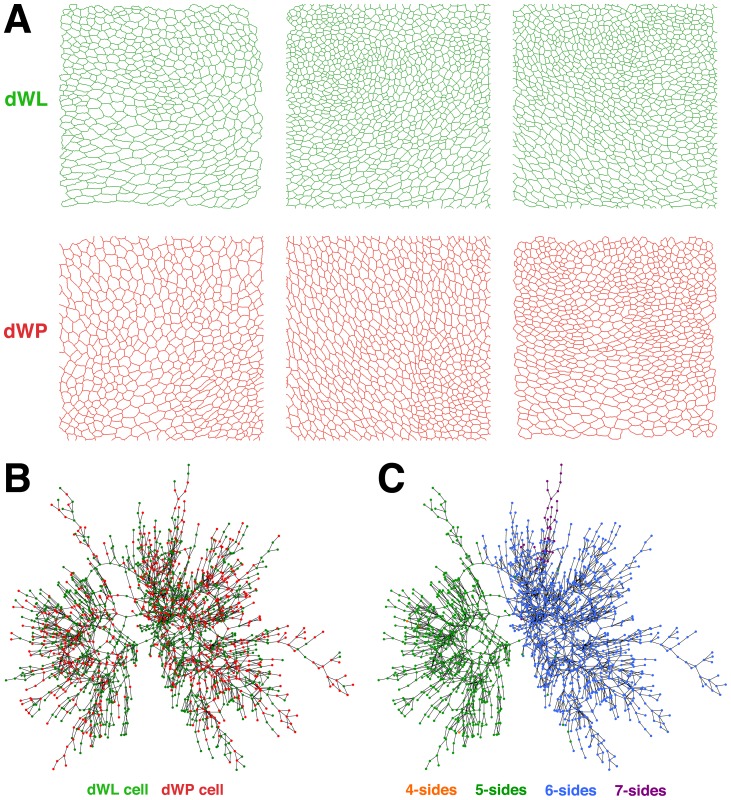
Epithelial images and cell correlation. **A**) Segmented image of three dWL (green) and dWP (red) images showing the extreme similarity between them. **B**) Visualization of the results of the correlation between dWL (green) and dWP (red) cells. The network contains cells from both types of images and each cell is represented by a node. Two nodes are linked if they present a similarity bigger than a certain threshold. The network shown in the panel is the one with a higher number of nodes (1729 cells) using a threshold of 0.9975. **C**) Representation of the same network of panel **B**, showing the distribution of sides of each cell. Orange, green, blue and purple mark 4, 5, 6, and 7 sided cells respectively. The image shows the high tendency of cells with the same number of sides to be linked.

We have used *Drosophila* wing epithelium as a model to understand the mechanisms that rule epithelial organization. Our dataset consisted in 15 samples from middle third instar wing discs (dWL), and 16 samples from the same region of early prepupa wing discs (dWP). These two proliferative stages are separated by 24 h of development. We have used segmented images from our previous study [26] in order to identify the objects (cells) that compose them. This enables the obtaining of geometric and topological characteristics from every cell. During the passage from mid third instar to prepupa there are only small changes at the level of the apical surface of the wing disc cells. Therefore these images appear very similar by simple visual inspection (**Fig. 1A**).

In order to identify subtle differences between dWL and dWP cells, we have tested a series of 14 characteristics that could reflect differences at the cellular level between our two types of images (**Table 1**). 1 to 5 were geometric features of the cells, while 6 to 14 corresponded to topological features capturing different aspects of the relation of each cell with its neighbours. Topological features were extracted constructing a network of cells contacts; with the centroid of the cells being nodes and adjacent cells being connected [26] and **Methods**). Once that we extracted the values for these 14 characteristics, we performed a correlation analysis using all the epithelial cells (dWL = 9070 cells, dWP = 6881 cells). As a result, we obtained a matrix showing the degree of similarity between every pair of cells. This dataset was interpreted using a network representation. Cells with a correlation coefficient above a determined threshold were connected building a “network of correlating cells” (**Fig. 1B-C**).

**Table 1:** List of characteristics analyzed in this study.

CHARACTERISTICS		
epithelial cc	Name	cell cc
**1**	Average Area	**1**
**2**	S. D. Area	
**3**	Average major Axis	**2**
**4**	Average minor Axis	**3**
**5**	Average Relation Axis	**4**
**6**	S. D. Relation Axis	
**7**	Average Convex Hull	**5**
**8**	S. D. Convex Hull	
**9**	Average Neighbours	
**10**	S. D. Neighbours	
**11**	Average Relation Neighbours Area	**6**
**12**	S. D. Relation Neighbours Area	
**13**	Average Relation Neighbours major axis	**7**
**14**	S. D. Relation Neighbours major axis	
**15**	Average Relation Neighbours minor axis	**8**
**16**	S. D. Relation Neighbours minor axis	
**17**	Average Relation Neighbours relation axis	**9**
**18**	S. D. Relation Neighbours relation axis	
**19**	Average Relation Neighbours convex hull	**10**
**20**	S. D. Relation Neighbours convex hull	
**21**	Average Strengths	**11**
**22**	S. D. Strengths	
**23**	Average Clustering Coefficient	**12**
**24**	S. D. Clustering Coefficient	
**25**	Average Eccentricity	**13**
**26**	S. D. Eccentricity	
**27**	Average Betweenness Centrality	**14**
**28**	S. D. Betweenness Centrality	
**29**	Average Shortest Paths lengths	
**30**	S. D. Shortest Paths Lengths	
**31**	Radius	
**32**	Diameter	
**33**	Efficiency	
**34**	Pearson correlation	
**35**	Algebraic connectivity	
**36**	S_metric	
**37**	Assortativity	
**38**	Density	
**39**	Transitivity	
**40**	Modularity	

Table shows names of the 40 characteristics analyzed in the feature selection step by PCA descriptor (a description is included in the **Text S1**). The 40 characteristics can be classified into three types: geometrically related to the size and shape of cells (1–8), network characteristics of the cells (9–28) and network characteristics of the image (29–40). The network features capture information about the organization of the cells. The grey background marks the 14 characteristics used in the cell correlation assay (numeration is on the right side).

Our hypothesis was that if these 14 features were able to differentially characterize cells from one particular stage we should find clusters of dWL cells and clusters of dWP cells. This was not the case. The largest correlation graphs always contained a mix of dWL and dWP cells (independently what threshold was used) (**Fig. 1B**). Our cell correlation experiments suggest that dWL and dWP cells do not present characteristics that allow their separation depending on the developmental stage. However, despite the fact that “number of cell sides” was not one of the characteristics used for the correlation, the combination of dWL and dWP cells in the network presented a clear preference in the distribution that depended on the type of polygon (**Fig. 1C**). Therefore, the “number of cell sides” bias the values for the 14 characteristics analyzed giving a certain local constrain to the tissue. These results encouraged us to search for differences at the next level, the polygon distribution.

### *Drosophila* wing epithelia change the polygon distribution during larval development

**Figure 2:**
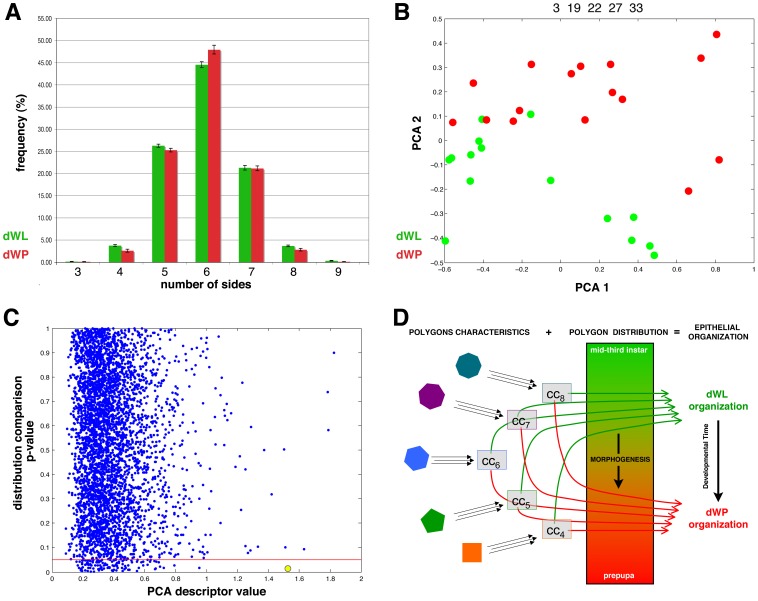
Epithelial organization differences between dWL and dWP. **A**) Polygon distribution of dWL (15 datapoints, green) and dWP (16 datapoints, red) images. The frequency of each type of polygons in both sets of images is represented. The error bars represent the standard error **B**) PCA graph for the comparisons of dWL (green dots) and dWP (red dots) images using the selected characteristics (numbers 3, 19, 22, 27 and 33). **C**) Graph representing the 4000 random combinations of images (blue dots). The *p*-value resulting from the MANOVA test of the distribution comparison is plotted against the PCA descriptor value of the same random combination. The red line marks the *p* = 0.05. The graphs show the absence of correlation between both values. The yellow circle marks the dWL-dWP combination. Only four combinations present a higher PCA descriptor value. None random combination with *p* ≤0.05 shows a PCA descriptor greater than dWL-dWP combination. **D**) Model for control of tissue organization during the end of the proliferative larval stage. The morphogenetic signals in the wing disc drive a change in polygon distribution between mid-third instar larva and early prepupa. Our results also support the existence of two separable organizations in each of these developmental time-points. The model propose that the number of sides of the cells imposes geometric and organizational local constraints that in combination with a determined variation of the percentage of each polygon propagate in a change of the tissue epithelial organization.

Previous studies have shown that the apparition of a determined polygon distribution in the wing disc of *Drosophila* (around 3% tetragons, 28% pentagons, 46% hexagons, 20% heptagons) is an inherent property of the proliferating epithelia and is present in other metazoan [17]. We calculated the percentage of cells with different number of sides in the two developmental stages analyzed (dWL and dWP; **Fig. 2A**). The average values were in the same range to the previously published [17], [22]. However, we found a small but significant difference between dWL and dWP polygon distribution (MANOVA test, *p* = 0.013, **Fig. 2A**). For example, dWL presented a lower number of hexagons and a higher number of pentagons than dWP. Hence, we conclude that developmental factors drive a polygon distribution variation during this proliferative period. One of these developmental factors could be the reduction of proliferation rate that occurs at the end of the larval stage [14], [15], [29].

### Fast developmental changes in epithelial organization are revealed by network characteristics of groups of cells

An arising question was if the change in polygon distribution might lead to a progression in epithelial organization that could be detected using independent approaches. In a recent work, we have shown that differences in epithelial organization can be captured using “network” characteristics stemming from images with a large number of cells [26]. These topological characteristics are extracted from the network of cellular contacts described before where every cell of the epithelium is considered a node that is connected with the adjacent cells [26]. Aiming to improve our previous method, we have designed a new set of 40 characteristics (**Table 1**). 32 of these characteristics comprised concepts from graph theory and complex networks [27]. After this, we used Principal Component Analysis (PCA) to analyze the results. PCA is an unbiased mathematical algorithm that lowers data dimensionality, such that it projects the data on orthogonal axes maximizing their dispersion. In our case the data are the feature vectors which represents the images under study. Thus, images can be plotted in a bi-dimensional space representing the two first principal component. An analysis of the components plots can then be used to identify similarities and differences between images. In this way, very different images will distribute far from each other, while similar images will cluster together. This graph allows the quantification of the differences between the groups formed for each type of data (dWL and dWP in our case). We have designed a feature selection step to obtain only the most relevant characteristics to discriminate between two types of images ([28] and **Methods**). This feature selection step performed different combinations of the 40 characteristics and tested which resulted in a best separation in the PCA graph. To calculate the degree of separation we used a “PCA descriptor” that gave us a numeric value for each PCA graph resulting from different combinations of characteristics (in our case from 0.1 to 1.8 approximately, see **Methods**). Therefore, the aim is to find the combination of characteristics that maximize this “PCA descriptor”. In the case of dWL-dWP comparison the program selected one geometric and four “network” features: “Average major Axis”, “Average Relation Neighbours Convex Hull”, “Average Betweenness Centrality”, “Standard Deviation Strength” and “Efficiency” (see **Discussion** and **Text S1**). Using these characteristics the PCA graphs showed dWL and dWP images separated with only a small region of overlapping (**Fig. 2B**). The corresponding value for the PCA descriptor was 1.527.

This result suggested that our approach was able to identify topological differences between these two developmental stages. However, one possibility was that our feature selection step was able to force the separation between both sets (resting biological relevance to the experiment). To discard this possibility, we designed an assay to generate random combinations of our images, perform the feature selection step, and calculate the PCA descriptor value (see **Methods**). The results for 4,000 loops showed that the value obtained for dWL-dWP comparison was higher than 99.9% of the random combinations (**Fig. S1**). This latter result supported the existence of real (and detectable) differences between dWL and dWP images.

### Relation between the change in epithelial polygon distribution and the emergence of a different organization

So far, we have shown that dWL and dWP images present a different polygon distribution and a different epithelial organization. It might be possible that the latter was simply a consequence of the significant variation in polygon distribution. Under the light of our experiments, this is not the case. First, we obtained the polygon distribution from the 4,000 random combinations used previously and performed a MANOVA test for each combination. We found that only in 32 cases (0.08%) the achieved *p*-value was lower than the obtained in the dWL-dWP comparison (**Fig. S2**). Second, we plot the *p*-values of the MANOVA test and the PCA descriptor results (**Fig. 2C**). The graph showed that there was not a general correlation between these two parameters: We found that the 4 cases with a better value for the PCA descriptor did not present differences in the polygon distribution. On the other hand, the value of the PCA descriptor for the arbitrary groups with bigger differences in their polygons distributions (MANOVA, *p*-value <0.05) were always smaller than 1. In summary, both features seem to be independent from each other.

Consequently, only in the case of the comparison dWL-dWP a high value of the PCA descriptor corresponded to a low *p*-value of the MANOVA test (**Fig. 2C**) suggesting the existence of developmental mechanisms that links polygon distribution changes with detectable organizational transformations.

## Discussion

The analysis of small global changes during development is key to understand the mechanisms that couple growth and morphogenesis. We have used the well-studied *Drosophila* wing disc epithelia as a test-tube, comparing geometric and topological properties of mid-third instar larva and early prepupa stages, two time-points without recognizable organizational patterns (**Fig. 1A**). The acquisition of the hexagonal packing leading to the final wing organization has been described as dependent on planar cell polarity genes during pupa stages [19]. Therefore, the last hours of larval development has been classically seen as an actively growing stage lacking coordinated rearrangements beyond the maintaining of a specific polygonal distribution [17]. Our results suggest the existence of small but significant topological differences between mid-third instar larva and prepupa stages, marking the onset of the reorganization at the proliferative phase.

We started searching differences at the level of the individual cells. Using a correlation approach we aimed to compare the similarities between all the cells that integrated our two groups of images. The similarity analysis produced a clear result: nodes (cells) were not grouped depending of the original developmental stage (**Fig. 1B**). On the contrary, the mapping was completely related to the number of sides of the cells: Six side cells were preferentially linked to other hexagons, pentagons were clustered together and also groups of heptagons were favoured (**Fig. 1C**). This experiment raises a clear conclusion; the values of the 14 characteristics used in the correlation (**Table 1**) were strongly dependent on the number of sides of the cells (although “number of sides” was not one of the 14 characteristics used), and independent of the developmental stage. This dependence affected not only geometric, but also topological characteristics of the individual cells. On the other hand, we found that the polygon distributions of dWL and dWP where significantly different. Putting together these two findings we hypothesize that if the number of sides of a cell influence its local topological characteristics, a significant alteration in the polygon distribution can be converted into a coordinated morphogenetic transformation (**Fig. 2D**). In other words, changes in the prevalence of a polygon over other could lead into the modification of the organization of the whole epithelium. However, although we favour this option, we cannot totally discard that both phenomena are interdependent and that a modification of the arrangement of the tissue would also lead to the change in polygon distribution.

The remaining question is if only a difference in polygon distribution can explain changes in epithelial organization. We have tested 4000 combinations of arbitrary groups of images to conclude that polygon distribution and epithelial organization are, therefore, independent features in the random combinations. Consequently, only our biologically meaningful combination was significant for both tests and better than other 4000 combinations (**Fig. 2C**). For this reason, we interpret that during development the change in polygon distribution that occurs between mid-third instar larva and early prepupa is highly coordinated. This enables the emergence of higher magnitude organizational changes that now can be captured with our new network based method.

These organizational changes are best described by the relevant characteristics used to differentiate dWL and dWP samples. The “Average Major Axis”, “Average Relation Neighbours Convex Hull” and “Average Betweeness Centrality” stand out as the more relevant when analyzing the order in which they appear in the feature selection step and their weights in the PCA. Interestingly, these three characteristics where also used in the correlation experiment. What is the biological meaning of them? Their definition can explain the changes during these last 24h of larval development. In the case of geometric characteristics such average major axis, it is easy to interpret that dWP cells growth increasing the value of their major axis (the same happen with the values for minor axis” and “Average area”, **Table S1**). The case of “Average Relation Neighbours Convex Hull” is more complicated. The convex hull is defined as the smallest convex set that contains the shape. However, to have a reference with respect the cell, we computed this value as “Area of cell / convex hull area of the cell”. Therefore, a value close to 1 indicates that the cell presents a convex shape with smooth and straight sides. An increase of wiggles and irregularities in the perimeter of the cell decreases this value. Our data indicate that the outline of dWP cells is more regular: the values of “Average Convex Hull” and “Average Relation Neighbours Convex Hull” are closer to 1 (Table S1). We interpret that in dWP images the cells are more similar to their neighbours in terms of contour regularity, and this is one of the most important features to discriminate between dWL and dWP. In biological terms, this characteristic could express in some extend the effect of the mechanical stress that stretch the cells outlines. Clusters of early prepupa cells would present higher tensions induced by the morphogenetic movements that occur at this stage [29]. The third more discriminant feature is the “Average Betweenness Centrality” defined by the fraction of all shortest paths in the network that contain a given node. Nodes with high values of betweenness centrality participate in a large number of shortest paths, they are usually called hubs [30], [31]. dWL present a greater number of these hubs combined with others cells with lower value. This suggests that the tissue is more heterogeneous in terms of connectivity of their nodes, i.e. organization of their cells. On the other hand, early prepupa samples show a smaller value of “Average Betweenness Centrality” and “Std. Dev. Betweenness Centrality”. These results, translated to our images, indicate an increase of the homogeneity of this tissue with respect to dWL. The remaining two characteristics present a lower PCA weight and complement the previous three to obtain a higher PCA descriptor value. Average Efficiency and Std. Dev. Strength would be related with the increase of homogeneity and size respectively. To summarize, the relevant characteristics able to separate dWL and dWP images indicate that the enrichment of six-sided cells starts when the disc is still growing. An enlargement of the cells, a smoothened of their outlines and an increase of the homogeneity of the whole epithelium lead to the epithelial hexagonal lattice that will be formed later at pupa stage [19]. Our new methods of analysis mark the onset of the “hexagonal packing” during the proliferation stage of larval development, although it only will became obvious at the pupa stage [19].

All our results together combine in a simple model (**Fig. 2D**). We have demonstrated that the number of sides of the cells imposes local geometric and organizational constraints. Therefore changes in the percentage of each polygon can propagate altering the whole epithelial organization. Surprisingly, only the variation in polygon distribution driven by morphogenesis leads to progression in epithelial organization (**Fig. 2C**). The question remains about how the coupling of growth and organization is orchestrated. A simple hypothesis could imply the planar cell polarity genes acting before cell proliferation is arrested. Also, it could be caused by topological changes induced by the decrease of proliferation rate observed at the end of the larval period. Other possibility, related to the newly proposed integration of force-sensing and signaling pathways [32], could also explain small organizational changes. Future works should shed light on the detailed mechanisms that drive the process.

Here we have presented a new method to discriminate between very similar epithelial images that identify the relevant characteristics that allow the separation. This approach can be easily adopted for the analysis of epithelia in other systems, and could be very valuable to analyze small developmental changes visualized using *in vivo* imaging.

## Materials and Methods

### Image analysis and extraction of characteristics

dWL (mid third instar) and dWP (early prepupa) images were obtained at 96h and 120h of development respectively (growth at 25°C). All images analyzed in this study come from [26]. A complete description of how the images were obtained can be found in the methods section of that article. In each image a Region of Interest (ROI) was established in order to exclude cells closer to the border of the image. The features of the cells falling within the ROI were calculated. The cells outside were only used in order to provide neighbours to the cells analyzed [26]. A network of cellular contacts was created taking the centroid of each cell as a node that links to centroids of adjacent cells. To build the network, we followed the same method described in [26] with only one modification: We have used a radius r = 4 for the circle used to identify the neighbours of each cell.

15951 cells have been analyzed in 31 images. We defined 14 features related with geometric and topological properties of the individual cells than can be extracted from them. These values were used in the correlation assay. We also defined 40 features of the images. The values for features 1 to 28 were computed extracting the value for each cell and calculating the average and the standard deviation of all the cells inside the ROI for each image (**Table 1** and **Text S1**) [26]. The remaining twelve characteristics where extracted directly from the network formed by the nodes inside the ROI. After the extraction of the characteristics, the values were normalized to enable the comparison between different characteristics.

### Correlation assay

We have performed a correlation assay to measure the similarity of the individual cells contained in our images. We have compared the values of 14 characteristics: “average (av.) area”; “av. major axis”, “av. minor axis”; “av. relation axis”; “av. convex hull”; “av. relation (rel.) of neighbours area”; “av. rel. of neighbours major axis”; “av. rel. of neighbours minor axis”; “av. rel. of neighbours relation axis”; “av. rel. of neighbours convex hull”; “av. rel. strengths”; “av. clustering coefficient”; “av. eccentricity”; “av. betweenness centrality”. The first step was the extraction of these 14 values from each one of 15951 cells, then, an exhaustive pair wise comparison was performed. Our correlation process compared cells along 14 features. If our data is defined as cell1 =  [feature 1, feature 2, …, feature 14] and cell2 = [feature 1, feature 2, …, feature 14], the following equation provides the correlation value of these two cells:
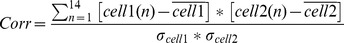



Where 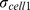 show us the standard deviation of cell1 data and 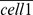 is the average of the normalized values of the 14 features from cell1. This measurement was done for each pair of cells. The obtained “Corr” value must be in a range between 0 and 1; the closer to one, the higher similarity between cells. We defined a threshold to get pairs of cells that are clearly similar. The threshold was applied as follow:

- If Corr (cell1,cell2)> = Threshold; then distance(cell1,cell2) = 1 -> Cells connected.
- If Corr (cell1,cell2)<Threshold; then distance(cell1,cell2) = 0 -> Cells disconnected.

Visone software was used to visualize the relation between cells. This program integrated the distance data to build the “correlation network” of connected cells. Two cells were connected if their correlation value was above the threshold. To determinate an optimum threshold value we analyzed the networks created. If threshold was too high, networks would present only few related cells. In the other hand, if threshold was too low, the resulting network would be difficult to provide some information. We examined different thresholds to corroborate that our results did not depend of the chosen limit. For clarity, we selected to show a threshold that created a larger network with 1500-2000 cells (**Fig. 1** **B, C**). Visone can manage different “Labels” that facilitates the analysis of the distribution of different characteristics of the cells.

### Randomization

In this work we have analyzed 31 images: 15 from mid third instar larva (dWL) and 16 from prepupa stage (dWP). Our results are based in the comparison of both groups. In some of our experiments we have randomly created new pairs of groups. We separated our 31 images in two groups: mixA (8 images from dWP and 8 images from dWL) and mixB (the 8 remaining images from dWP and the 7 images from dWL). For each randomization loop, two new mixA and mixB groups were obtained and analyzed. Finally, we have compared the results obtained with the original dWL and dWP groups with the 4000 randomizations.

### Polygon distribution analysis

To evaluate if the polygonal distribution of two groups of images was significantly different a Multivariate Analysis of Variance (MANOVA) was used. If *p-value* <0.05, distributions were considered to be significantly different. We compared the polygon distribution of dWL and dWP groups and 4000 random combinations of our 31 images. We performed all these MANOVA tests using the values for cells with 4, 5, 6, 7 and 8 sides. We discarded the values for the cells with 3, 9 and 10 sides, since they were not present in all the images.

### Principal Component Analysis (PCA) and PCA's Descriptor

We have used PCA [33] to analyze the differences between our two sets of images using the extracted characteristics [26]. PCA transforms the correlated data points of the feature vector into a small number of uncorrelated variables called principal components. The projection maximises the dispersion of the individual data points in an unbiased way. This allows the identification of naturally separated sets of data points (images in our case). These data points can be visualized graphically based on its position on the PCA graph when 2 principal components are represented. Once the PCA graph was obtained, we used a variant of Calinski-Harabasz descriptor to evaluate the degree of separation between 2 groups of images [34].
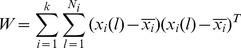


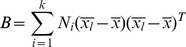


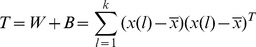


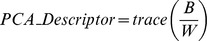



Where it is given a set 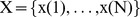 of N data objects and a partition of these data into *k* mutually disjoint cluster, 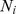 is the number of objects assigned to the 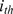 cluster, 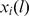 is the 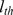 object assigned to that cluster, 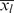 is the n-dimensional vector of sample means within that cluster (cluster centroid) and 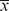 is the n-dimensional vector of overall sample means (data centroid). As such, the within-group and between-group matrices sum up to the scatter matrix of the data set [33], [34]. As a consequence, compact and separated clusters are expected to have small values of W and large values of B. Hence, the better the data partition the greater the value of the ratio between B and W [34].

### Features selection by PCA descriptor

We have defined an iterative method for the selection of the relevant features (among our 40 characteristics) that distinguish better two groups of images. The method selects and evaluates features using the descriptor explained above. The method tests every possible combination of two features and applies the PCA. The method keeps the ten combinations of two features with higher PCA descriptor value. In the second iteration, all features are individually tested again in combination with the ten couples of two features. Again, all the combinations are evaluated and the program keeps the five with higher PCA descriptor value for each one of the ten couples. Therefore, at this moment the program handles 50 trios of features. In the next iteration, the same process is repeated but only two best features are added, accumulating 100 quartets of features. The process continues adding only one feature per iteration step. Finally, the process is stopped when seven features have been selected or when the value for the PCA descriptor is lower than in the previous step. The selected combination of features is the one with a higher PCA descriptor value.

## Supporting Information

Figure S1 **Randomization of images combinations and their respective PCA descriptor values.** Graph showing the number of combinations with a determined PCA descriptor value. Only four combinations present a PCA descriptor value higher than dWL-dWP combination (yellow square).
(TIF)

Figure S2 **Randomization of images combinations and the MANOVA test p-value for their respective polygon distribution.** Graph showing the number of combinations with a determined *p*-value for the MANOVA test. The polygon distribution for each combination was compared using the MANOVA test. The number of cases with a determined *p*-value is represented. The yellow square corresponds to the *p*-value of the combination dWL-dWP.
(TIF)

Table S1 **Values for the 40 characteristics analyzed in the 31 epithelial images.** The table shows both real and normalized quantities after the extraction of the values for the 40 characteristics. Yellow boxes highlight the characteristics selected after the PCA descriptor feature selection step. The bottom part graph compares the normalized values of dWL (green) and dWP (red) for each characteristic.
(XLSX)

Text S1 Supporting Definitions.
(DOC)
