# Supplementary figures and images for: Topological Progression in Proliferating Epithelia Is Driven by a Unique Variation in Polygon Distribution
Source: PLoS One. 2013 Nov 5;8(11):e79227. doi: 10.1371/journal.pone.0079227 (PMC3818423; doi:10.1371/journal.pone.0079227)

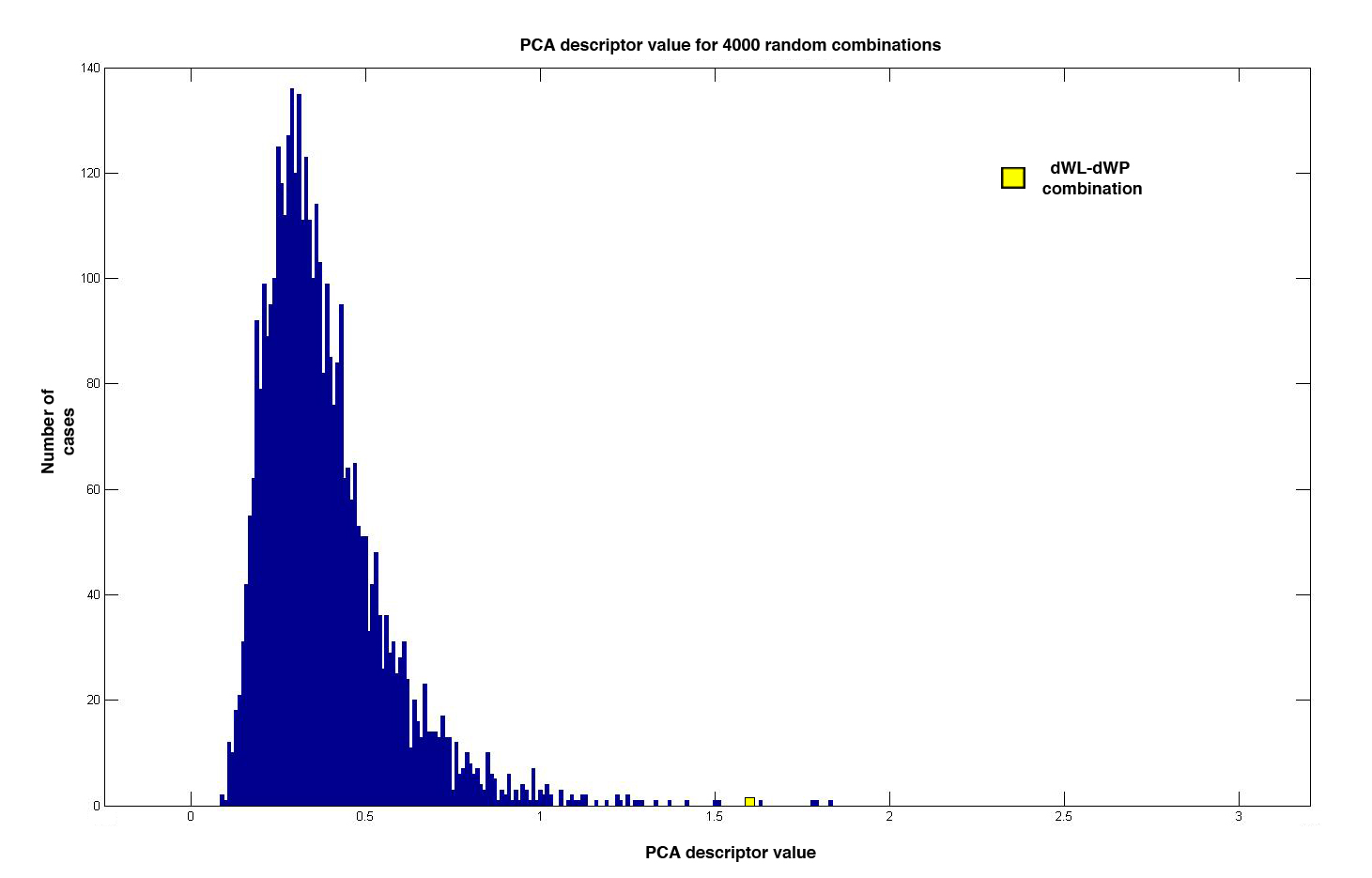

Supplement: Figure S1 — Randomization of images combinations and their respective PCA descriptor values. Graph showing the number of combinations with a determined PCA descriptor value. Only four combinations present a PCA descriptor value higher than dWL-dWP combination (yellow square). (TIF) [file pone.0079227.s001.tif]

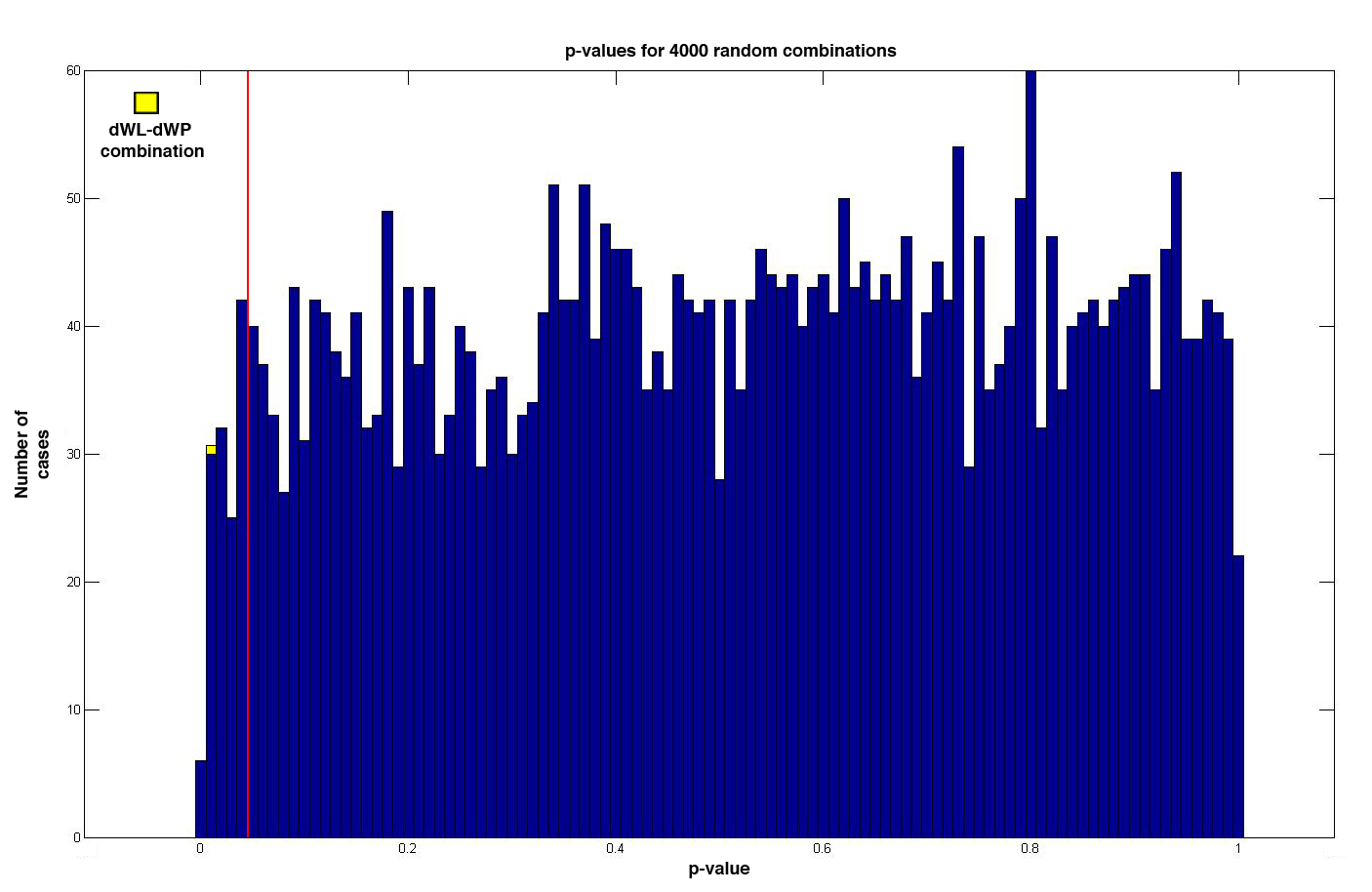

Supplement: Figure S2 — Randomization of images combinations and the MANOVA test p-value for their respective polygon distribution. Graph showing the number of combinations with a determined p-value for the MANOVA test. The polygon distribution for each combination was compared using the MANOVA test. The number of cases with a determined p-value is represented. The yellow square corresponds to the p-value of the combination dWL-dWP. (TIF) [file pone.0079227.s002.tif]
