# Supplementary material for: Topological Progression in Proliferating Epithelia Is Driven by a Unique Variation in Polygon Distribution
Source: PLoS One. 2013 Nov 5;8(11):e79227. doi: 10.1371/journal.pone.0079227 (PMC3818423; doi:10.1371/journal.pone.0079227)
Supplement: Text S1 — Supporting Definitions. (DOC) [file pone.0079227.s004.doc]

**Topological progression in proliferating epithelia is driven by a unique variation in polygon distribution.**

**Daniel Sánchez-Gutiérrez, Aurora Sáez, Alberto Pascual, Luis M. Escudero.**

**SUPPORTING DEFINITIONS**

Definitions of the 40 characteristics analyzed in this study.

***Area*:** Size (in pixels) of the cell.

***Major Axis*:** Length (in pixels) of the major axis of the ellipse that has the same normalized second central moments as the cell.

***Minor Axis*:** Length (in pixels) of the minor axis of the ellipse that has the same normalized second central moments as the cell.

***Relation Axis*:** Ratio between the major and minor axes of each cell.

***Convex Hull*:** Proportion of the pixels in the convex hull that are also in the cell. Computed as Area of cell/Area of the convex hull. The convex hull is the smallest convex polygon that can contain the cell.

***Neighbours*:** Number of neighbours cells of a cell.

Characteristics 11 to 28 are related to the value for a geometric characteristic of a node and the average value of its neighbours. A short description is given for characteristics 29 to 40:

***Strength***: Node strength is the sum of weights of links connected to the node, where the weight of links, in our case, is the distance in pixels between two cells.

***Clustering coefficient***: The fraction of triangles around a node (equivalent to the fraction of a node’s neighbours that are neighbours of each other).

***Eccentricity:*** The shortest path length between a node and any other node.

***Betweenness centrality***: The fraction of all shortest paths in the network that contain a given node. Nodes with high values of betweenness centrality participate in a large number of shortest paths.

***Shortest path lengths***: The distance matrix containing lengths of shortest paths between all pairs of nodes.

***Radius:*** The minimum eccentricity.

***Diameter:*** The maximum eccentricity.

***Efficiency:*** The average inverse shortest path length in a network.

***Pearson correlation***: The Pearson correlation reflects the degree of linear relationship between two variables (nodes and weight of links).

***Algebraic_connectivity***: The second smallest eigenvalue of the Laplacian (Laplacian: degree matrix minus the adjacency. Adjacency matrix: matrix with rows and columns labeled by graph nodes, with a 1 or 0 in position (v_i_, v_j_) according to whether v_i_ and v_j_ are adjacent or not).

***S_metric***: The sum of products of degrees across all edges.

***Assortativity***: A positive assortativity coefficient indicates that nodes tend to link to other nodes to the same or a similar degree.

***Density***: The fraction of present connections to possible connections. Connection weights are ignored in calculations.

***Transitivity***: The ratio of 'triangles to triplets' in the network (an alternative version of the clustering coefficient).

***Modularity:*** A statistic that quantifies the degree to which the network may be subdivided into such clearly delineated groups.
